# Knowledge, attitudes and practices about dengue among pupils from rural schools in Colombia

**DOI:** 10.7705/biomedica.4255

**Published:** 2019-09-01

**Authors:** Diana Sarmiento-Senior, María Inés Matiz, Juan Felipe Jaramillo-Gómez, Víctor Alberto Olano, Sandra Lucía Vargas, Neal Alexander, Audrey Lenhart, Thor Axel Stenström, Hans Jörgen Overgaard

**Affiliations:** 1 Instituto de Salud y Ambiente, Universidad El Bosque, Bogotá, D.C., Colombia Universidad El Bosque Instituto de Salud y Ambiente Universidad El Bosque BogotáD.C Colombia; 2 MRC Tropical Epidemiology Group, London School of Hygiene and Tropical Medicine, London, United Kingdom MRC Tropical Epidemiology Group London School of Hygiene and Tropical Medicine London United Kingdom; 3 Liverpool School of Tropical Medicine, Liverpool, United Kingdom Liverpool School of Tropical Medicine Liverpool United Kingdom; 4 Institute for Water and Wastewater Technology, Durban University of Technology, Durban, South Africa Institute for Water and Wastewater Technology Durban University of Technology Durban South Africa; 5 Faculty of Science and Technology, Norwegian University of Life Sciences, Ås, Norway Faculty of Science and Technology Norwegian University of Life Sciences Norway

**Keywords:** Dengue/prevention and control, health knowledge attitudes practice, rural health, ethnology, Colombia, dengue/prevención y control, conocimientos, actitudes y práctica en salud, salud rural, etnología, Colombia

## Abstract

**Introduction:**

Dengue is a public health problem in tropical and subtropical regions of the world. Studies on dengue in rural areas are scarce since the disease is considered mainly urban.

**Objective:**

To determine the knowledge (K), attitudes (A) and practices (P) of dengue in an endemic area in Colombia.

**Materials and methods:**

We conducted a cross-sectional study with 515 pupils (7-16 years old) in 34 rural schools in Anapoima and La Mesa municipalities during 2011. Each KAP category was evaluated independently by a scoring system and then categorized into high, medium or low.

**Results:**

Pupils recognized knowledge variables such as the symptoms (fever, bone pain), transmission route (mosquito bites), and mosquito breeding sites (uncovered wáter tanks, solid waste). Average scores on attitude were high in both municipalities indicating a well-developed perception of disease severity. Seeking treatment in medical centers and self-medication for fever management and the use of mosquito net and space-spraying of insecticides were the most frequently identified practices.

**Discussion:**

This is the first KAP dengue study performed in a rural area in Colombia and as such it contributes to the understanding of dengue perceptions by the inhabitants of these areas. It showed a medium level of knowledge about dengue and a lower level of preventive practices in pupils from rural schools. It also showed that pupils considered space-spraying as crucial for vector control. The presence of the vector in rural areas of the country underlines the need to improve surveillance and education to more effectively control the vector and promote prevention methods including community participation.

## Resumen

**Introducción.:**

El dengue es un problema de salud pública en las regiones tropicales y subtropicales del mundo. Los estudios sobre la enfermedad en áreas rurales son escasos debido a que se presenta principalmente en los núcleos urbanos.

**Objetivo.:**

Determinar los conocimientos, las actitudes y las prácticas sobre el dengue en un área rural endémica en Colombia.

**Materiales y métodos.:**

Se hizo un estudio transversal con 515 estudiantes, entre los 7 y los 16 años de edad en 34 escuelas rurales de los municipios de Anapoima y La Mesa en el 2011. Cada categoría se evaluó por separado con un puntaje clasificado como alto, medio o bajo.

**Resultados.:**

Los estudiantes reconocieron los síntomas (fiebre, dolor de huesos), la ruta de transmisión (picadura de mosquitos) y los criaderos (depósitos de agua destapados, residuos sólidos). El promedio de los puntajes de actitudes fue alto en ambos municipios, lo que indica que percibían la gravedad del dengue. Las prácticas más frecuentes de tratamiento de la fiebre fueron la consulta médica y la automedicación, en tanto que los métodos de control más frecuentes fueron el uso de mosquiteros y el rociamiento con insecticidas.

**Discusión.:**

Este es el primer estudio de conocimientos, actitudes y prácticas sobre el dengue en un área rural de Colombia. Los resultados contribuyen a entender las percepciones de la enfermedad en sus habitantes y evidenciaron niveles medios de conocimiento y puntuaciones bajas en cuanto a las prácticas entre los estudiantes de las escuelas rurales. Los estudiantes consideraron que la fumigación es importante para el control del vector. La presencia del mosquito en áreas rurales del país resalta la necesidad de mejorar la vigilancia y la educación para lograr un control efectivo del vector y promover métodos de prevención que incluyan la participación comunitaria.

Dengue is a serious and increasing public health problem in tropical and subtropical regions of the world ^1^ with approximately 3,900 million people at risk of infection ^2^, around 390 million annual infections, and 96 million presenting clinical signs ^3^. In 2010, Colombia experienced its worst-ever epidemic with more than 157,000 reported cases and 217 deaths. Anapoima and La Mesa municipalities in the department of Cundinamarca were classified as very high-risk areas based on their number of cases ^4^.

Among the determinants of the increasing incidence are the simultaneous circulation of four serotypes ^5^, the lack of continuous water supply, por or inadequate solid waste management, unplanned urban growth, internal displacement, and poverty ^4^. Besides, the situation is worsened by climatic variations such as El Niño and La Niña phenomena that can make conditions more favorable for the main vectors *Aedes aegypti* and *A. albopictus* and disease transmission ^4^. Recently, the circulation of other viruses such as chikungunya and Zika may have contributed to an underestimation of dengue through misdiagnosis due to their similarity of symptoms and the high cost of laboratory tests that hinder their application in every case ^6^.

Although the World Health Organization (WHO) recommended a vaccine as part of an integrated dengue prevention and control strategy ^7^, there is no dengue vaccination program in Colombia. The only preventive measures available are based on mosquito control, which is highly dependent on community participation ^8^. In recent decades, this has been increasingly recognized and acknowledged in studies on integrated dengue interventions linking local human populations and their practices on prevention and vector control ^9^^,^^10^.

The need to determine how knowledge about the disease is related to prevention practices has been highlighted in community and school guides and reports ^9^. However, information strategies do not always change behaviors, such as the elimination of domestic breeding sites ^11^, sometimes because recommended practices are neither well-suited to existing habits nor adapted to the socio-economic conditions of the communities ^11^^,^^12^.

Educational institutions can function as training centers and meeting sites for communities from which health education, productive projects, and new technologies can be disseminated ^8^^,^^13^^-^^16^. Some dengue prevention initiatives have used schools as points of community communication and assessed knowledge, attitudes, and practices (KAP) of pupils, their families or their communities ^14^^-^^16^. However, such KAP studies are scarce in rural areas, since dengue transmission is considered mainly urban ^4^^,^^10^. Nevertheless, *A. aegypti* is present in rural areas, sometimes with high virus infectivity suggesting active transmission ^5^^,^^17^.

The present study was part of the baseline work of a trial of integrated interventions for preventing dengue and diarrhea in rural Colombian schools ^18^. The objective was to determine the KAP about dengue among pupils in grades 2 to 5 in rural schools in Anapoima and La Mesa municipalities in Cundinamarca department.

## Materials and methods

**Figure 1:**
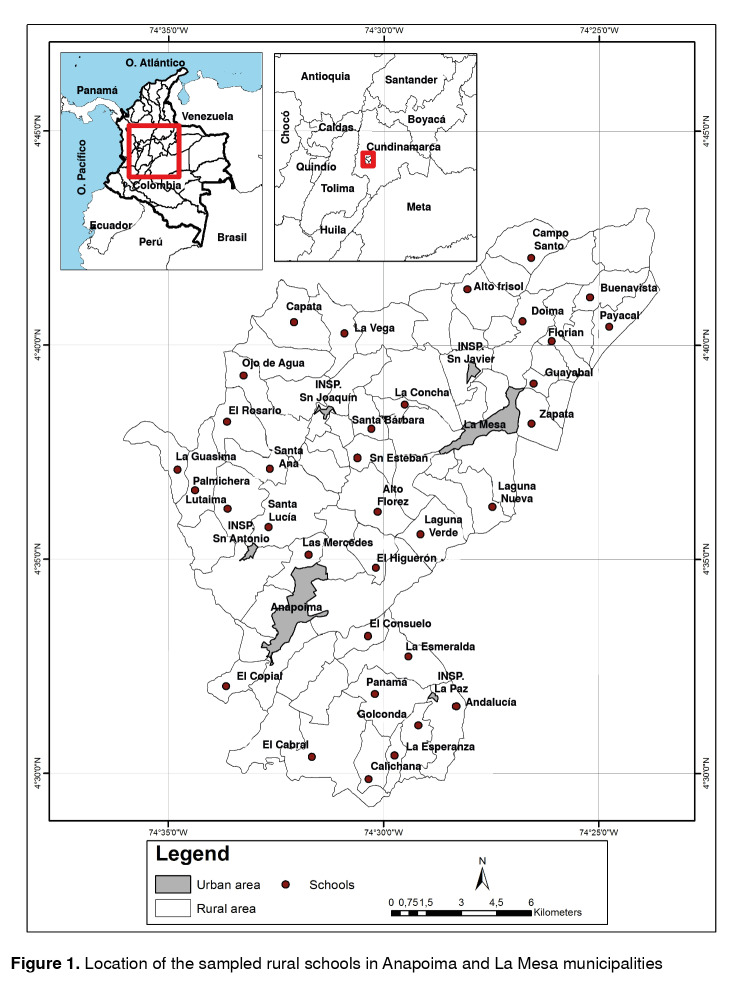
Location of the sampled rural schools in Anapoima and La Mesa municipalities

We conducted a cross-sectional study in rural schools (<100 pupils and ≤5 grades) of Anapoima and La Mesa, 17 schools in each municipality (figure 1); 828 pupils were eligible to participate in the study according to the records of the schools. KAP surveys were performed from May to June 2011 with 515 pupils in grades 2 to 5: 309 in La Mesa and 206 in Anapoima (table 1).

**Table 1:** Description of the pupils surveyed in Anapoima and La Mesa municipalities in 2011

Variable	Anapoima		La Mesa		Total	
	n	%	n	%	n	%
Total number of pupils in the study	206	40	309	60	515	100
Mean pupil age (in years) (SD)	9.4 (1.6)		9.3 (1.7)		9.3 (1.7)	
Age range (in years)	7-16		6-14		6-16	
Female population	86	41.7	138	44.7	224	43.5
Male population	120	58.3	171	55.3	291	56.5
2nd grade	51	24.8	74	23.9	125	24.3
3rd grade	47	22.8	86	27.8	133	25.8
4th grade	50	24.3	81	26.2	131	25.4
5th grade	58	28.2	68	22.0	126	24.5

### Study area

Anapoima has an average altitude of 700 meters above sea level, a mean temperature of 26°C, an area of 124.2 km², and a population of 12,539 inhabitants (56% rural) ^19^^,^^20^. La Mesa is 1,200 meters above sea level, its mean temperature is 22°C, it has an area of 148 km², and 29,566 inhabitants (45% rural) ^20^^,^^21^. The mean annual rainfall is 1,300 mm in both municipalities. 

The main economic activities are tourism and agriculture based on sugar cane, coffee, and fruit tree crops, as well as livestock. In 2010, the proportion of people with unmet basic needs (UBN), an indicator of poverty, in the rural areas of Anapoima was 36.4%, while in the urban areas it was 21.2% ^19^. In La Mesa, the proportions were 36.8% and 11.8%, respectively ^21^. Anapoima has a lower average altitude and a higher proportion of rural population. Nevertheless, La Mesa has a larger rural area. Both municipalities were classified as very high- risk areas based on their number of cases of dengue in the last 15 years ^4^.

### Data collection

A questionnaire in Spanish was developed, field-tested and reviewed by experts in education, epidemiology, and entomology. The language was chosen to optimize comprehension and clarity considering the target age group. A member of the project attended readings with groups of up to five pupils in grades 2 and 3. This person read and showed the questions and response options ensuring that all items were answered without inducing answers. Pupils in grades 4 and 5 read the questionnaire independently without intervention from other pupils or project staff. Two or more members of the project team accompanied each group, which had at most 15 pupils.

The questionnaire consisted of six multiple-choice questions, three for establishing knowledge about dengue, one for attitudes and two for prevention practices. Knowledge of the symptoms of dengue, its mode of transmission, and identification of four mosquito breeding sites (laundry tanks or *albercas* -large concrete tanks used for storing water for laundry, one of the most common breeding sites in the area-, tires, uncovered water tanks, solid waste) were evaluated. The question regarding attitudes related to disease severity. As for practices, the pupil was asked about fever management and measures for dengue prevention and control. The identification of practices for fever management by pupils was included in the practices section considering that children usually receive care from adults. Additional variables included age, sex, grade, and municipality. The questionnaire was delivered to pupils in the target age group (grades 2-5) present in the school who were able to read and write, without cognitive disabilities, and who had provided parental consent (oral or written) and personal assent (oral or written).

### Analysis

Individual scores were calculated for the knowledge, attitudes and practices sections and then scaled to a range of 0 to 10. Values for each student were used to calculate averages by school and municipality. For the knowledge and practice scales, categories were divided into quartiles and classified into three groups (high, medium, and low) by combining the two groups below the 50th percentile. For the knowledge scale, the low quartile was <6.6, the medium between 6.6 and 8.3, and the high >8.3. The corresponding figures for the scale of practices were <5.4, 5.4-7.2, and >7.3. A scale for attitudes was not developed because the only possible values were zero or ten.

To determine the homogeneity of variance, Fisher F tests were performed where if the p value was greater than 0.05, the assumption of equal variances was considered valid. Student’s t test for independent groups was used to compare mean scores by municipality, where a p value of <0.05 was considered statistically significant. The Huber-White estimator of the covariance matrix was used to allow for clustering within schools.

The KAP surveys data were entered and cleaned in Microsoft Excel™, version 2010, and analyzed in Epidat™, version 3.1, and R, version 3.1.1, including the package ‘rms’.

### Ethical considerations

Based on national regulations, the study was classified as minimal risk ^22^. The institutional research ethics committee of *Universidad El Bosque*, Bogotá, Colombia (Minutes No. 146 of 30/08/2 011) and the Ethical Review Board of the London School of Hygiene and Tropical Medicine (Ref. No. 10 453(6 289)-1) approved the study. Directors and teachers of the schools gave written consent. Written consent of parents and written assent were sought for all pupils enrolled. Assent was sought from pupils after it was explained that participating or not would have no academic or disciplinary implications. Only those giving oral assent participated in the survey.

## Results

### Description of the population

Out of the total population of pupils reported as enrolled in the rural schools, 62.2% (n=515) participated in the survey. Sixty percent of pupils in the sample lived in La Mesa municipality and the remainder in Anapoima. The average age was 9.3 years (SD=1.7), 9.4 for males and 9.2 for females. The majority were male with 58% in Anapoima and 55% in La Mesa. The largest group was the fifth grade in Anapoima and the third grade in La Mesa, each representing approximately 28% of the surveyed population (table 1).

### Knowledge

**Figure 2:**
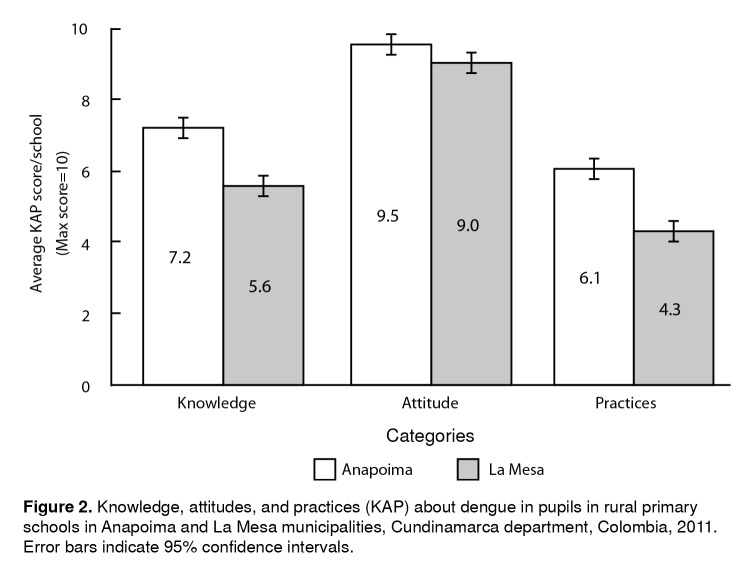
Knowledge, attitudes, and practices (KAP) about dengue in pupils in rural primary schools in Anapoima and La Mesa municipalities, Cundinamarca department, Colombia, 2011. Error bars indicate 95% confidence intervals.

The average knowledge score was significantly higher in Anapoima than in La Mesa, 7.2 (95% confidence interval [95% CI]: 6.9-7.5) and 5.6 (95% CI:5.3-5.9), respectively (t[513]= -8.0, p<0.001) (figure 2). In Anapoima, 3 of 17 schools reached a high-level score (above 8.3), and 9 of 17 schools reached the medium level. In La Mesa, just one school achieved a medium score while the remaining 16 schools were in the low category.

A set of manifestations for dengue disease such as fever, bone pain, red spots on the skin, and bleeding from the nose and gums were identified by 87 (17%) of the pupils. The most frequently recognized dengue symptoms were fever (56% La Mesa, 74% Anapoima) and bone pain (48% La Mesa, 64% Anapoima) (table 2). Mosquito bite was the transmission route selected by 87% of the pupils (85% La Mesa, 91% Anapoima). Overall, 116 (23%) of the respondents identified all the four options for potential breeding sites of the dengue vector. Considering both municipalities together, the most frequently identified breeding sites were uncovered water tanks (68%) and solid waste (59%). Eighty percent of the pupils in Anapoima and only 40% of pupils in La Mesa identified washbasins (*albercas*) as breeding sites (table 2).

**Table 2:** Number and percentage of pupils with knowledge about dengue symptoms, transmission routes, and mosquito breeding sites in rural schools in Anapoima and La Mesa municipalities, Cundinamarca department, Colombia, 2011

	Anapoima		La Mesa		Total	
	n=206	%	n=309	%	n=515	%
Symptoms						
Fever	152	73.8	173	56.0	325	63.1
Bone pain	131	63.6	148	47.9	279	54.2
Bleeding from the nose and gums	131	63.6	94	30.4	225	43.7
Red spots on the skin	128	62.1	92	29.8	220	42.7
Transmission route						
Mosquito bite	187	90.8	263	85.1	450	87.4
Proximity to someone sick	29	14.1	94	30.4	123	23.9
Via food	9	4.4	4	1.3	13	2.5
Breeding sites						
Uncovered water tanks	161	78.2	189	61.2	350	68.0
Solid waste	119	57.8	183	59.2	302	58.6
Washbasins (*albercas*)	165	80.1	124	40.1	289	56.1
Tires	141	68.4	100	32.4	241	46.8

### Attitudes

The overall perception of disease severity revealed that 92% of pupils (Anapoima 95%, La Mesa 90%) thought that dengue is a health problem for them. There was no significant difference between Anapoima and La Mesa, 9.5 (95% CI: 9.2-9.8) and 9 (95% CI: 8.7-9.3), respectively (t[504]= 1.9, p=0.16) (figure 2).

### Practices

The pupils from Anapoima had a significantly higher average score than those from La Mesa, 6.1 (95% CI: 5.8-6.4) and 4.3 (95% CI: 4-4.6), respectively (t[488]= -8.3, p<0.001) (figure 2). Anapoima’s average score was in the medium category while La Mesa’s was low. The main practices for fever management identified among the seven options given were seeking care at a medical center (Anapoima 82%, La Mesa 72%), and taking medication (Anapoima 69%, La Mesa 65%). Other practices that pupils identified were the use of mosquito nets and receiving herbal infusions (table 3).

**Table 3:** Number and percentage of pupils identifying practices for fever management and dengue prevention and control in rural schools in Anapoima and La Mesa municipalities, Cundinamarca department, Colombia, 2011

	Anapoima		La Mesa		Total	
	n=206	%	n=309	%	n=515	%
Practices for fever management						
Going to the medical center	169	82.0	222	71.8	391	75.9
Taking medication	142	68.9	200	64.7	342	66.4
Using mosquito net	108	52.4	89	28.8	197	38.3
Receiving herbal infusions	83	40.3	92	29.8	175	34.0
Bathing and refreshing	64	31.1	102	33.0	166	32.2
Drinking water and abundant liquids	66	32.0	88	28.5	154	29.9
Actions for dengue prevention and control						
Using mosquito net	170	82.5	195	63.1	365	70.9
Fumigation with insecticides	149	72.3	208	67.3	357	69.3
Washing *albercas*	162	78.6	142	46.0	304	59.0
Changing water of vases	160	77.7	138	44.7	298	57.9
Taking out water of tires	155	75.2	129	41.7	284	55.1
Covering water containers	140	68.0	145	46.9	285	55.3
Learning and teaching	28	13.6	23	7.4	51	10

a *Albercas*: large concrete tanks used for storing water for laundry

Among the actions to be taken to avoid the disease, the most common answer in both municipalities was the use of mosquito nets (Anapoima 83%, La Mesa 63%). Other practices considered important in La Mesa were space-spraying of insecticides and covering water storage containers while in Anapoima, respondents said washing the *albercas* and changing the water in floral vases (table 3).

## Discussion

In this study, we determined KAP among pupils in rural schools in Anapoima and La Mesa municipalities. Overall, we found a medium level of knowledge about dengue and a lower level of preventive practices in both municipalities. While schools, health institutions or media mainly promote knowledge, practices to control the vector depend on many factors such as infrastructure, socio-economic conditions, demographic characteristics, and personal choice ^23^^-^^25^.

Although the national surveillance public health policy defines activities for dengue prevention and control ^26^, the regular vector control strategies adopted by local health boards may differ between municipalities. During an epidemic, it is mandatory to stop transmission according to the national protocol; municipalities also carry out complementary actions such as solid waste collection campaigns in critical areas. Despite being neighbors, the two municipalities in this study differ in terms of altitude and proportion of rural areas and population, which may affect decisions on vector control strategies and the activities that are recognized by inhabitants. Anapoima experienced higher dengue incidence than La Mesa in 2010 (Anapoima: 3,198 per 100,000 population vs. La Mesa: 1,975 per 100,000 population) and in 2011 (Anapoima: 679 per 100,000 population vs. La Mesa 160 per 100,000 population) ^27^. Higher scores in knowledge and practices of pupils from Anapoima may have been influenced by the delivery of an educational intervention in the municipality for prevention and control of dengue with community agents in rural areas in 2010 ^28^, as well as municipal education campaigns as a response to the high incidence of dengue.

Nevertheless, these activities usually focused on urban areas ^18^.

Comparable studies in rural areas of Colombia are scarce since dengue is mainly considered an urban disease ^4^^,^^10^. However, greater attention has recently been given to the occurrence of the disease in rural areas showing vector presence, rural infections ^17^^,^^23^^,^^29^, and the presence of the virus in the vector ^5^. Our study, therefore, adds valuable new information on awareness and practices, which is relevant for the planning of disease prevention programs. However, a consensus needs to be reached on measurement methods for knowledge, attitudes, and practices to allow comparison of results.

### Knowledge

Thirty percent of the pupils in Anapoima identified a combination of disease manifestations compared to only 8% in La Mesa. Other studies conducted in urban primary schools in Latin America found that 77% of pupils in Honduras ^16^, 41% in Brazil ^13^, and 42% in México ^30^ identified dengue symptoms such as fever, bone pain, and red spots on the skin when asked what it feels like to have dengue. KAP studies can be compared across age and school grade, but results have to be interpreted carefully because of differences between populations, socioeconomic status, and health surveillance determining urban and rural conditions.

Eighty-seven percent of the pupils identified that the disease is transmitted by mosquito bite, similar to 92% in the Philippines ^23^ and 97% in Malaysia ^29^. Despite the young age of the pupils in this study, their knowledge of this aspect exceeded two previous Latin American studies, which found that 68% of pupils surveyed in Honduras ^16^ and 56% in México ^30^ knew that mosquitoes transmit dengue. Better identification of the mode of transmission may be related to the strengthening of mass media messages in recent years ^8^. Moreover, actions for prevention and control carried out by public health programs and entomological surveillance have increased ^26^. Inhabitants of these areas are in constant contact with the disease, which facilitates learning from their own experiences or through contact with health staff ^12^. Overall, 24% of the pupils said that dengue can be transmitted by proximity to someone who is sick, a high proportion compared to 14% reported by pupils in an urban location in Colombia ^15^. Few KAP studies on dengue report other answers than mosquito bites being the main route of transmission. However, qualitative approaches have identified that some populations think of the disease as an everyday illness or associate it with common cold ^12^.

Just 23% of the pupils in our study recognized all the four given breeding sites, in contrast to 68% of pupils in Honduras ^16^ and 49% in México ^30^ asked to identify three. Only 40% of pupils in La Mesa identified the *alberca* as a breeding site, although this is a common *A. aegypti* breeding site ^12^^,^^31^. Their elimination as breeding sites is hindered by their large volume and lack of an efficient way to seal them ^31^. Only 61.2% of rural areas in Colombia have access to regular piped water supply ^32^, which forces inhabitants to store water in containers potentially providing breeding sites for the vector. Some studies identified a lack of accuracy in the knowledge related to breeding places and more appropriate water storage methods as important themes for community education to focus on ^25^.

A majority of pupils in our study identified solid waste as a breeding site for the mosquito that transmits dengue. We found that 59% of the pupils associated solid waste with the mosquito while a study conducted in Brazil showed that 78% of the general population did so ^25^. Where appropriate rubbish collection practices and programs are lacking, people dispose of waste in open fields.

### Attitudes

Pupils from both municipalities expressed a high level of concern regarding the risk of dengue. Pupils in La Mesa recognized the risk to a slightly lesser extent (90%) than the ones in Anapoima (95%), although the difference was not statistically significant. Our results are similar to those of other studies, which found that dengue and its risks are a matter of concern for communities ^25^^,^^33^. Measuring attitudes related to the disease should be strengthened, and questionnaires should be supplemented with qualitative approaches ^11^^,^^12^^,^^25^^,^^30^.

### Practices

The average scores of practices were significantly higher in Anapoima than in La Mesa. We asked pupils about fever management practices of adults. Even though pupils were not directly guiding these practices due to their age, we asked them to identify the typical procedures undertaken by their parents and caregivers. They reported seeking help at a medical center (76%) as a fever management practice similar to results from comparable studies in Latin America ^29^^,^^33^. Strategies like home care or self-medication ^12^ were the second most common response, which is reasonable considering existing poor roads, scarce means of transport, availability of health systems in rural areas, difficulties in accessing medical services, and parents’ consideration that illness is not severe enough to seek help at a medical center ^12^^,^^25^. Although seeking help at a medical center was the most frequent answer, it has been shown that some families choose home care at the beginning of the disease ^12^.

Our study also included domestic fever management practices such as herbal infusions (34%) and fluid administration (30%). These have seldom been included in other studies ^11^. Mass-media campaigns generally focus on information about community vector control, although there is a real need to improve knowledge about the disease and home care of infected persons ^34^.

A notable finding is the relatively low percentage of pupils that identified the use of mosquito net as a prevention measure during fever episodes (overall 38%). The use of mosquito nets for dengue prevention is generally low in Colombia although health authorities promote their use by sick people in endemic areas to avoid onward dengue transmission ^10^. In fact, national health authorities in Colombia recommend the use of mosquito nets as an individual strategy to prevent the transmission of the disease in households and hospitals during the first eight days of fever ^35^. However, this did not seem to be clearly recognized in our study. Data from urban areas showed that 6% in Carepa and Apartadó (Antioquia department) ^34^ and 28% in Bucaramanga (Santander department) ^33^ reported using nets against dengue. This is different from a rural area in the Philippines, where 59% of the population used nets for protection against dengue ^23^. The emphasis of educational and vector control interventions on container management rather than home care could be a reason for the low recognition of this strategy ^34^^,^^36^.

Space-spraying of insecticides was the second most common choice for prevention. Several studies have shown that inhabitants perceive space-spraying as crucial for vector control ^12^^,^^24^^,^^34^^,^^36^. The intensive use of insecticides by health authorities can hinder the implementation of other preventive interventions because it can lead communities to downplay their responsibility or the impact of their practices in prevention ^24^. The ranking of control methods identified in La Mesa could reflect the familiarity of the pupils with these procedures, as well as the low identification of other prevention methods.

Overall, 55% of pupils in our study recognized that covering water containers is a strategy to prevent and control dengue. We consider this percentage to be low given how central the activity is to dengue control. Sixty- five percent of pupils in Medellín ^15^ and 72% of pupils in Puerto Rico ^14^ identified breeding site reduction as a dengue intervention.

Considering the weaknesses found in previous interventions, education plans adapted to community interests and resources are needed ^12^^,^^15^^,^^25^. Participatory approaches from the design stage of the intervention can motivate people to get involved in changing risk behaviors. Education from an early age is a key factor in people’s understanding of the disease and the implementation of risk-reducing practices ^24^^,^^36^.

Where appropriate, the school curriculum should include these educational processes acknowledging rural conditions. This may lead to applicable preventive measures and play-based strategies ^14^^-^^16^^,^^33^. When assessing knowledge gaps, it is important to make distinctions based on, for example, age, educational level, and urban/rural settings. The distinction between academic and applicable knowledge in the everyday lives of rural inhabitants and the relationship to socio-economic constraints must be acknowledged.

It is necessary to focus on rural residents’ concepts of illness, awareness of the disease, use of space-spraying only in emergencies, and actions, especially self-medication and use of mosquito nets for home care of children, infants and the sick in general. Despite having a basic knowledge of the disease, the individuals and the community have yet to fully transform this knowledge into protective practices. Hence, it is imperative to link academia and the government to strengthen community action through the improvement of the water, sanitation and health infrastructure, as well as the innovative use of resources and capacities for long-term inter-sector interventions ^34^^,^^36^.

Few KAP studies have been performed in rural Colombia since dengue is mainly considered an urban disease. To our knowledge, this is the first dengue KAP study in a rural area in the country. The recent evidence of the presence of *A. aegypti* and the virus in rural areas requires improved entomological and epidemiological surveillance, as well as educational programs oriented to both pupils and community members.

Good knowledge does not guarantee its transformation into better practices, especially if there is a lack of stimuli, insufficient water, sanitation and health infrastructure development, and low community participation.

Pupils demonstrated a good knowledge of the transmission route of dengue while knowledge of symptoms and breeding sites was mixed indicating the need to strengthen this knowledge from an early age. Results on practices showed ambivalence on the use of mosquito nets and low percentages identifying the covering of water containers as a preventive strategy, also, a high tendency for self-medication. There remains a need to measure preventive practices in rural areas and identify remaining barriers to their implementation.
